# Cardiotoxicity as important differential diagnosis for reduced myocardial blood flow during Rubidium cardiac PET/CT: Cardiotoxicity in Rubidium PET/CT

**DOI:** 10.1007/s10554-024-03315-4

**Published:** 2025-01-08

**Authors:** Sabin G. Pop, Eva Hägler, Cristina Popescu, Irene A. Burger, Alexander W. Sauter

**Affiliations:** 1 Department of Nuclear Medicine, Cantonal Hospital Baden, Partner Hospital for Research and Teaching of the Medical Faculty of the University of Zurich, Baden, 5404 Switzerland; 2 Department of Cardiology, Cantonal Hospital Baden, Partner Hospital for Research and Teaching of the Medical Faculty of the University of Zurich, Baden, 5404 Switzerland; 3 Department of Nuclear Medicine, University Hospital Zurich, University of Zurich, Zurich, 8006 Switzerland; 4 Department of Radiology, University Hospital Tuebingen, Tuebingen, 72076 Germany

**Keywords:** Cardiotoxicity, Positron emission tomography computed tomography, Rubidium-82, Myocardial flow reserve, Chemotherapy, Breast neoplasms

## Abstract

A 65-year-old woman with a history of ductal mammary carcinoma and recent autonomic dysfunction underwent a Rb-82 chloride (RbCl) cardiac PET/CT scan that showed no ischemia or scarring, but significantly reduced myocardial flow reserve (MFR) (global: 1.5) and a CAC-Score of 0. The patient’s chemotherapy history (paclitaxel, carboplatin, epirubicin, pembrolizumab 2 years before) with elevated Troponin T and NT-pro-BNP levels at that time, and now reduced MFR with 0 CAC suggests cancer-therapy-related cardiotoxicity. An important differential diagnosis to the more common CAD-associated microvascular disease. Furthermore, tumor recurrence with a PET-avid lymph node metastasis was found additionally.

**Fig. 1:**
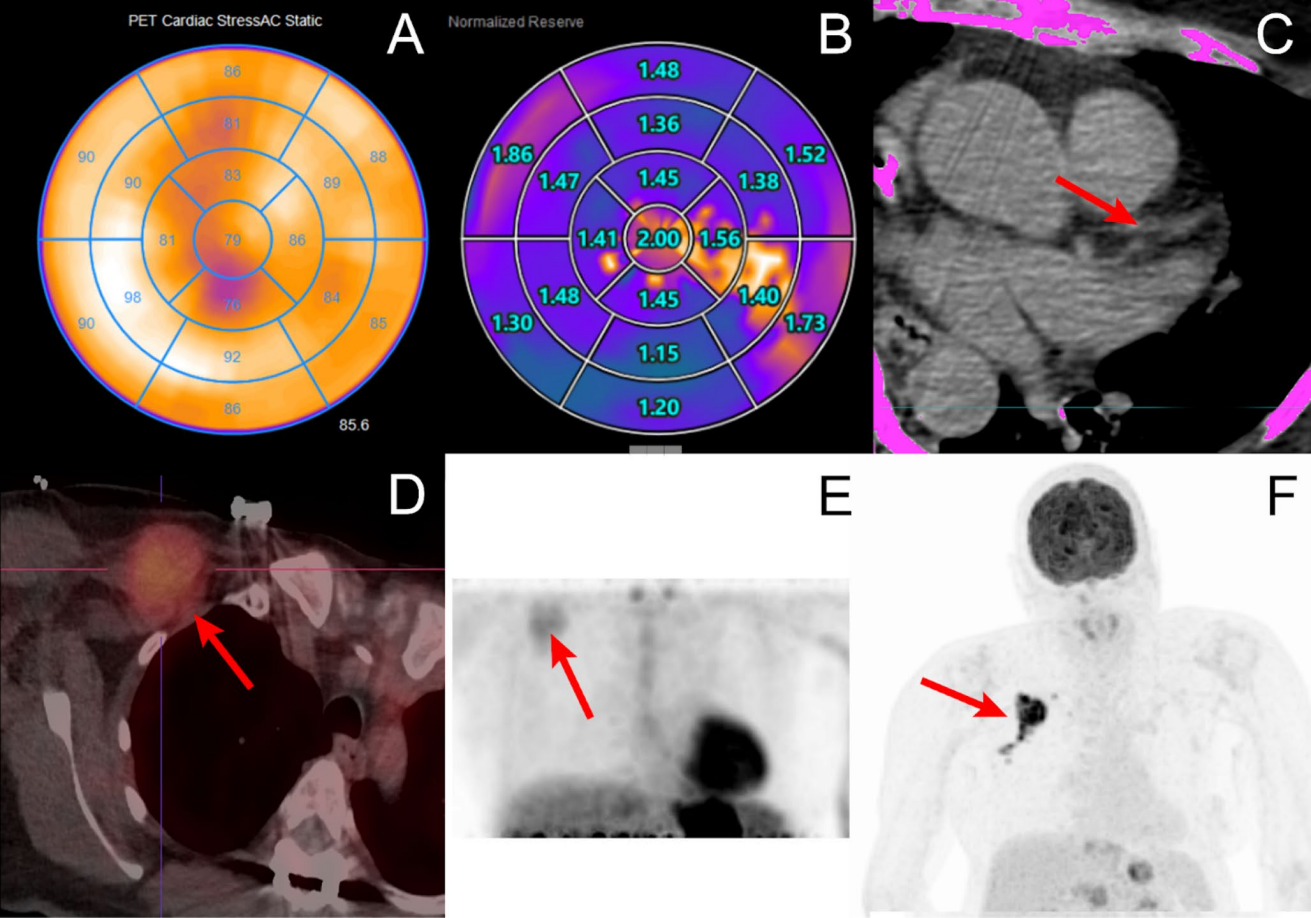
A-E illustrates findings from a Rb-82 chloride (RbCl) cardiac PET/CT scan of a 65-year-old woman, recently diagnosed with autonomic dysfunction and a history of ductal mammary carcinoma. The examination showed no evidence of ischemia or scarring (Fig. 1.**A**), but revealed a significantly reduced coronary flow reserve (Global: 1.5; Normal range > 2.0) (Fig. 1.**B**). Despite this, the coronary calcium score (CAC-Score) was 0 (Fig. 1.**C**), leading to a diagnosis of coronary microvascular disease (CMD). Furthermore, the scan identified an avid lesion in the right subpectoral region, suspicious for a lymph node metastasis of the ductal carcinoma (Fig. 1.**D** and **E**) [1], later confirmed as a triple-negative breast cancer recurrence using FDG-PET/CT (Fig. 1.**F**) and histology

Advances in chemotherapy and immune checkpoint inhibitors have significantly improved cancer survival rates [2]. However, these treatments, depending on the type of drug, dosage, and duration, can lead to myocardial damage and subsequent cardiotoxicity [3, 4]. Myocardial perfusion imaging using positron emission tomography (PET) is a non-invasive technique that quantifies the myocardial blood flow (MBF) under rest and stress conditions and measures the myocardial flow reserve (MFR). With the increasing use of these technologies and improved survival rates in cancer patients, cancer-therapy-related cardiotoxicity has become a more observed issue, highlighting the need for greater awareness within the medical community.

In our case, while the PET/CT findings are suggesting coronary microvascular disease, it’s crucial to consider the clinical context. The patient, first diagnosed with ductal mammary carcinoma in 2022, underwent four cycles of paclitaxel and carboplatin, followed by four cycles of epirubicin/cyclophosphamide combined with pembrolizumab. After an unilateral mastectomy in September 2022 additional 9 cycles of pembrolizumab were applied. Despite being asymptomatic, the Troponin T and NT-proBNP levels raised in autumn 2022 (74.2 and 757 ng/ml, respectively), although the echocardiography and electrocardiography showed no abnormalities with stable ejection fraction (EF) and global longitudinal strain.

Given these findings, and considering the chemotherapy history, a diagnosis of chemotherapy-induced cardiotoxicity is an important differential diagnosis. Interpreting the reduced MFR without clinical information might lead to the differential diagnosis of microvascular dysfunction related to coronary artery disease (CAD). However, the more likely diagnosis here would be CMD attributed to chemotherapy-induced cardiotoxicity, a condition that can precede structural heart changes without EF reduction, but elevated cardiac enzymes. Noteworthy, the patient management of the 2 entities is completely different.

Epirubicin, especially combined with trastuzumab, is known for its cardiotoxic effects, but only 11.1% of the enrolled patients in a randomized controlled trial showed an EF drop and only 1.5% cardiac heart failure [5]. However, EF reduction is at a later stage of toxicity and more subtle changes that initially affect the microvasculature might be underestimated [6]. Finally, CMD is an independent predictor of cancer development and it is therefore important to report reduced MFR as a potential pre-cancerous risk factor [7]. This might support the thesis of CMD as an early sign of cancer [8].

## Data Availability

No datasets were generated or analysed during the current study.

## Abbreviations

CAC-Score: Coronary calcium score
CMD: Coronary microvascular disease
PET: Positron emission tomography
MBF: Myocardial blood flow
MFR: Myocardial flow reserve
EF: Ejection fraction
CAD: Coronary artery disease
